# Particle-Scale Modeling to Understand Liquid Distribution in Twin-Screw Wet Granulation

**DOI:** 10.3390/pharmaceutics13070928

**Published:** 2021-06-22

**Authors:** Ashish Kumar, Stefan Radl, Krist V. Gernaey, Thomas De Beer, Ingmar Nopens

**Affiliations:** 1 Pharmaceutical Engineering Research Group (PharmaEng), Department of Pharmaceutical Analysis, Faculty of Pharmaceutical Sciences, Ghent University, Ottergemsesteenweg, B-9000 Ghent, Belgium; 2 Institute for Process and Particle Engineering, Graz University of Technology, Inffeldgasse 13/3, 8010 Graz, Austria; radl@tugraz.at; 3 Process and Systems Engineering Center (PROSYS), Department of Chemical and Biochemical Engineering, Technical University of Denmark, 2800 Kongens Lyngby, Denmark; kvg@kt.dtu.dk; 4 Laboratory of Pharmaceutical Process Analytical Technology, Department of Pharmaceutical Analysis, Faculty of Pharmaceutical Sciences, Ghent University, Ottergemsesteenweg, B-9000 Ghent, Belgium; Thomas.DeBeer@Ugent.be; 5 BIOMATH, Department of Mathematical Modelling, Statistics and Bioinformatics, Faculty of Bioscience Engineering, Ghent University, Coupure Links 653, B-9000 Ghent, Belgium; Ingmar.Nopens@UGent.be

**Keywords:** discrete element method, wet granulation, pharmaceutical processing

## Abstract

Experimental characterization of solid-liquid mixing for a high shear wet granulation process in a twin-screw granulator (TSG) is very challenging. This is due to the opacity of the multiphase system and high-speed processing. In this study, discrete element method (DEM) based simulations are performed for a short quasi-two-dimensional simulation domain, incorporating models for liquid bridge formation, rupture, and the effect of the bridges on inter-particular forces. Based on the knowledge gained from these simulations, the kneading section of a twin-screw wet granulation process was simulated. The time evolution of particle flow and liquid distribution between particles, leading to the formation of agglomerates, was analyzed. The study showed that agglomeration is a rather delayed process that takes place once the free liquid on the particle surface is well distributed.

## 1. Introduction

An accurate description of aggregation and breakage rate expressions is of great interest for the high shear wet granulation research and development. Specifically, this interest is fueled by the need for rational process design, i.e., to produce granules with the desired quality and maximize yield. Mechanistic modeling is seen as a powerful tool to develop this understanding regarding the wet granulation process and equipment design. Many mechanistic expressions, i.e., so-called *rate kernels*, have also been developed in this regard, describing aggregation and breakage rates [1]. However, these expressions often require the specification of different material properties, such as wetness, porosity, and yield strength. The mesoscale frameworks like population balance equations (PBEs) typically require closures for (i) collision rates, (ii) particle collision velocities, or data on other particle-scale phenomena. Additionally, material fluxes (i.e., the axial and radial velocities) need a suitable description for successful dynamic simulation of the mixing process. Unfortunately, the particle-scale information for these closures is known to be dependent on process parameters, equipment geometry, or material properties that cannot be directly computed. Hence, a common strategy is to calibrate these unknown parameters required in the closures using experimental data [2]. Despite calibration, such closure models have limited applicability due to the lack of predictive capabilities outside of their experimental domain [3]. This is unsatisfactory, especially if one aims for process optimization or change the granulator’s geometry to improve the performance.

In contrast, the discrete element method (DEM) is a micro-scale framework that tracks individual particles, or granules, as they move through space and time. This highly-accurate physical modeling tool can provide details on the flow of particles or granules. This information, e.g., collision rates and velocity profiles, can then be fed into the rate kernels, incorporating effects due to current process parameters, the equipment geometry, and material properties. Based on the level of detail, this can further be classified as particle-scale and granule-scale simulations, finding different applications.

The “particle-scale” simulations can be used for extensive investigation of particle-scale phenomena such as mixing of entities since particles in a granular assembly are treated as a separate entity and tracked. Previous attempts of particle-scale simulations include the work of Lian et al. [4] and McCarthy [5]. However, this also makes such simulations considerably more expensive than the granule-scale DEM-based simulations as a large number of entities (i.e., particles) have to be tracked.

DEM-based simulations that track individual granules (i.e., ensembles of particles) are frequently used [3,6,7]. This type of simulation is referred to as “granule-scale” since phenomena on the scale of the granules, typically with a size of 100 to 1000 µm, can be directly simulated. The advantage of these simulations is that a comparably small number of entities (i.e., granules) have to be tracked, and that granules are allowed to break or aggregate. This opens the door to investigate the effect of the actual geometry of a twin-screw granulator (TSG). Also, variations in granule properties, such as porosity and liquid content, are often accounted for in this type of DEM simulations using empirical correlations for, e.g., the coefficient of restitution or Young’s modulus [8]. Closures for mechanisms such as aggregation, breakage, and consolidation, which cause changes in particle size and other properties, need to be provided in granule-scale DEM simulations. Therefore, often a multi-scale approach is suggested where DEM data is collected and used within a population balance model (PBM) by either one-directional or bi-directional coupling [7]. Most of the multi-scale studies, such as Gantt et al. [9] used one-directional coupling of DEM with PBM to evaluate mechanistic coalescence kernels. Goldschmidt et al. [10] used DEM simulations to solve a PBM, replacing small particles with larger ones as they successfully coalesce. Bouffard et al. [11] used DEM to evaluate a spatial transport in a compartmental PBM. Additionally, Reinhold and Briesen [12] developed a coupled PBM-DEM model for wet granulation in which DEM simulations were used to evaluate a mechanistic aggregation rate kernel. Dhenge et al. [13] first used DEM to investigate surface velocity of powder in a TSG with conveying screws. Recently, Zheng et al. [14] used a GPU-enhanced DEM simulation for investigating the transport of dry particle systems through a TSG. Barrasso et al. [3] implemented a bi-directional coupling between PBM and DEM to evaluate collision frequencies and liquid distribution as a proof-of-concept. The model showed sensitivities to the screw configuration, process parameters such as screw speed, liquid-to-solid ratio, and material properties such as binder viscosity and pore saturation. However, this bi-directional coupling of a PBM-DEM model for wet granulation depends on the assumption that liquid is distributed evenly with respect to particle volume, which is not the case as per experimental studies [15]. Recently, Tamrakar et al. [16] integrated the complex interactions such as powder wetting behavior, capillary and viscous liquid bridge formation as well as binder dissolution within DEM to simulate two binder addition approaches—wet and dry, during high shear wet granulation (HSWG). However, particle level effects, like breakage or attrition, were omitted from the DEM model. In fact, achieving a homogeneous distribution of liquid, which largely controls the constitutive mechanisms of the twin-screw wet granulation, is the primary requirement for driving the granulation process towards a required state [17]. Therefore, investigating the drivers for particle and liquid mixing in the TSG is key to our understanding of the twin-screw wet granulation process.

In our present study to investigate particle and liquid mixing in the mixing zone of a TSG, our focus was first to track individual particles in a short quasi-two-dimensional simulation domain. Our simulations incorporate detailed models for liquid-bridge formation, rupture, as well as the effect of the bridges on inter-particular forces. Thereafter, the same simulation approach was applied to the mixing zone of a TSG, and the effect of process parameters on particle and liquid mixing rate was investigated.

## 2. Particle Scale Modeling Approach

### 2.1. Particle Flow Model

**Figure 1:**
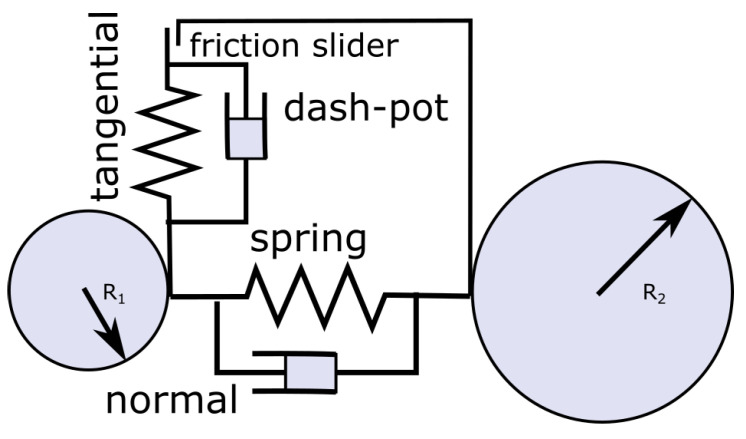
Representation of normal and tangential contact forces using a spring, dash-pot and slider approach.

Computer simulations using the DEM were performed using the software package LIGGGHTS [18]. In a dense particle system forces acting on a particle can be decomposed into a body force $F_{i}^{b}$ on particle *i*, as well as into a contact $F_{ij}^{con}$ and cohesion force $F_{ij}^{coh}$ between particles *i* and *j*. The interaction of the granular flow with the surrounding air has been neglected in our work due to the high particle fill ratio and the large particle-to-air density ratio. The so-called soft-sphere method, in which collisions are modeled as enduring contacts, was used. The hypothetical overlap between particles, occurring over a few time steps, is used to model the particles’ deformation. Contact forces are a function of these overlaps. The forces are expressed with the use of a spring, dash-pot and slider model, which separate forces into normal and tangential forces, as shown in Figure 1. Based on these forces, the trajectories of individual particles were computed using Newton’s equation of translational and rotational motion.

The contact force $F_{ij}^{con}$ acting on particle *i*, from particle *j* can be decomposed into a normal $F_{i}^{c,n}$ and a tangential component $F_{i}^{c,t}$, which are modelled as:

(1) $$F_{i}^{c,n}=k_{n}\delta n_{ij}-\eta_{n}v_{ij}^{n}$$

(2) $$F_{i}^{c,n}=-k_{t}\delta u_{ij}^{t}-\eta_{t}v_{ij}^{t}$$

where $u_{ij}^{t}$ is the tangential overlap and δ is the normal overlap between particles *i* and *j*:

(3) $$\delta =r_{i}+r_{j}-\left|r_{i}^{*}+r_{j}^{*}\right|$$

By Newton’s third law, particle *j* experiences the same contact force as particle *i* but in the opposite direction. The characteristic contact time is $t_{c}$ given by

(4) $$t_{c}=\pi /\omega$$

where

(5) $$\omega =\sqrt{k_{n}/m_{\mathrm{eff}}-\eta_{n}^{2}/\left(4m_{\mathrm{eff}}^{2}\right)}$$

This contact time sets the time step Δt for the integration of Newton’s equation of motion. The restitution coefficient $e_{n}$, which is the ratio of relative velocities of the particle after and before collision, is approximated as:

(6) $$e_{n}=\exp\left(\frac{-\pi \eta_{n}}{m_{\mathrm{eff}}\sqrt{4k_{n}/m_{\mathrm{eff}}-\eta_{n}^{2}/m_{\mathrm{eff}}^{2}}}\right)$$

to calculate the damping $\eta_{n}$.

### 2.2. Liquid Bridge Model

When the granulation liquid, denoted as liquid phase in what follows, is added to dry powder particles in the TSG, the surface of the particles is wetted, and the liquid on the particle surface is distributed between the particles in contact. Liquid distribution in the moving dense particle system progresses due to (i) liquid transfer facilitated by the particle-particle collisions and (ii) the convective transport of the particle motion by throughput force of incoming material and drag force of the rotating screws. Depending on the ratio between solid and liquid volume, different wetting states are achieved [19]. The cohesive forces between the wetted particles strongly depend on the exact shape of the liquid bridge and the pressure difference between the gas and liquid phase. The stability of a liquid bridge between two particles and its rupture thus mechanistically dictates the macroscopic granulation status. For simulating cohesive forces, the capillary force model proposed by Mikami et al. [20] was used. In the case of bridge formation, model C proposed by Mohan et al. [21] was used for tracking the liquid distribution. In this model, when wet particles touch each other, a certain fraction of the liquid on the particle surface $\phi_{tr}$ was exchanged at a finite rate $Q_{tr}$ calculated as

(7) $$Q_{tr}{}_{,i}=-\frac{c_{f}}{t_{\mathrm{ref}}}\left(L_{p,0}\phi_{tr}-V_{b}\right)$$

where $c_{f}$ is a dimensionless filling rate coefficient and $t_{\mathrm{ref}}$ is the reference liquid bridge filling time calculated as

(8) $$t_{\mathrm{ref}}=\frac{r_{\mathrm{eff}}\mu_{l}}{\sigma_{l}},\;\mathrm{with}\;r_{\mathrm{eff}}=\frac{2r_{i}r_{j}}{{\left(r_{i}+r_{j}\right)}^{2}}$$

and,

(9) $$\phi_{tr}=1-\sqrt{1-\frac{r_{j}^{2}}{{\left(r_{i}+r_{j}\right)}^{2}}}$$

For a more detailed discussion regarding the liquid transfer model see the study by Mohan et al. [21].

#### Liquid Loading, Bridge Volume Fraction & Liquid Bridge Coordination Number

In a TSG, liquid is added to a region of the granulator which is later distributed by the shearing motion of the particles. The amount of liquid load on the particles in the liquid addition region was calculated as

(10) $$Q_{lod}=\frac{Q_{i}}{\frac{\pi }{6}n_{p,liq}d_{p}^{3}}t_{\exp}$$

At the steady state condition, the liquid bridge volume is given by

(11) $$V_{b}=\frac{1}{2}\left(L_{p}{}_{i}+L_{p}{}_{j}\right)\phi_{tr}$$

The global-average fraction of liquid in liquid bridges relative to the total amount of liquid in the system, i.e., the liquid bridge fraction, was calculated as:

(12) $$V_{bf}=\frac{\sum_{i=1}^{n_{p}}V_{b}{}_{i}}{\sum_{i=1}^{n_{p}}\left(V_{b}{}_{i}+L_{p}{}_{i}\right)}$$

As the liquid tracking model assesses the per-particle liquid content, the number of liquid bridges and particle-particle contacts at each time step was also tracked. This information can be used to compute $n_{b,i}$ i.e., the average number of liquid bridges per particle as:

(13) $${\bar{Z}}_{b}=\frac{2\sum_{i=1}^{n_{p}}n_{b,i}}{n_{p}}$$

### 2.3. Simulation Set-Up and Input Parameters

#### 2.3.1. Simple Periodic Simulation Box

**Figure 2:**
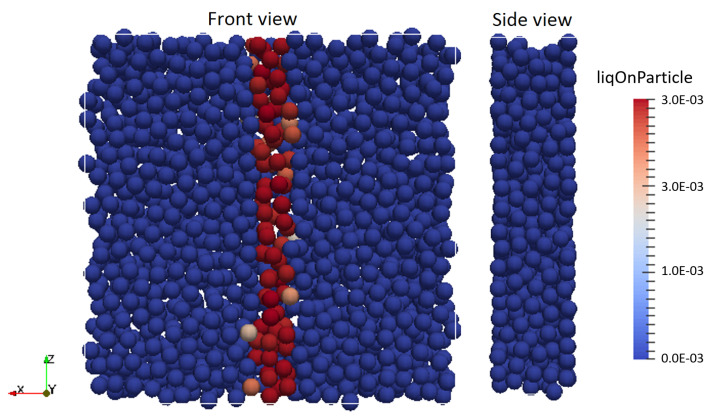
Shear box initial condition, front and side views. The red coloured particles indicate the liquid addition region (i.e., the wetting zone).

In order to investigate the solid-liquid mixing under well-controlled flow conditions, an assembly of about 1500 mono-disperse particles (the exact number varied based on the fill ratio) of diameter $d_{p}$ and density $\rho_{p}$ were placed in a periodic box of equal length and width ($H/d_{p}=20$) at particle volume fractions ranging from 0.3 to 0.5 (see Figure 2 for a typical setup). A homogeneous shear flow, with the shear gradient pointing in the *x*-direction, was applied based on Lees–Edwards boundary conditions [22]. The *y*-direction is the spanwise direction. The reference data for the rheology in this set-up is available without wall effects because each point in the simulation domain is statistically identical. The dimensionless shear rate (Equation (14)), which is defined as the ratio of a characteristic deformation time of the particles and a characteristic shearing time, was used to characterize the shearing rate:

(14) $$\gamma^{*}=\gamma d_{p}^{3/2}/\sqrt{k_{n}/\rho_{p}}$$

For soft particles, $\gamma^{*}$ $>10^{-1}$ [23], thus the simulations were carried out for a fixed $\gamma^{*}$ of 1.

A gradient of liquid content on the particles was imposed in the middle zone of 2$d_{p}$ width in the periodic box, both in positive and negative *y*-directions. The liquid content of the particles in this zone was fixed, while keeping other particles dry (i.e., liquid content and bridge volume equals zero). The liquid content on the particles was defined based on a dimensionless reference film thickness ϵ = $h_{0}$/$r_{\mathrm{eff}}$ where, $h_{0}$ is proportional to the reference liquid content on the particles via $L_{p,0}$ = $d_{p}\pi h_{0}$. A typical snapshot of such a simulation at the initial state (i.e., time $t_{0}$) is presented in Figure 2. The material properties applied in these simulations are based on measurements on lactose particles by Perkins et al. [24] and using water as a granulation liquid. The simulation parameters are listed in Table 1. The spring stiffnesses and damping coefficients in normal and tangential direction were calculated from these parameters considering the LIGGGHTS User Manual for the chosen contact model [25]. The timestep size during simulations was selected to correspond to the timestep of 20% of the Rayleigh time criterion, $dt_{r}$ calculated as:

(15) $$dt_{r}=\pi r\frac{\sqrt{\rho_{p}/G}}{0.1631\nu +0.8766}$$

where $\rho_{p}$ is particle density, *G* is the shear modulus and ν is Poisson’s ratio.

#### 2.3.2. Mixing Zone of a TSG

**Figure 3:**
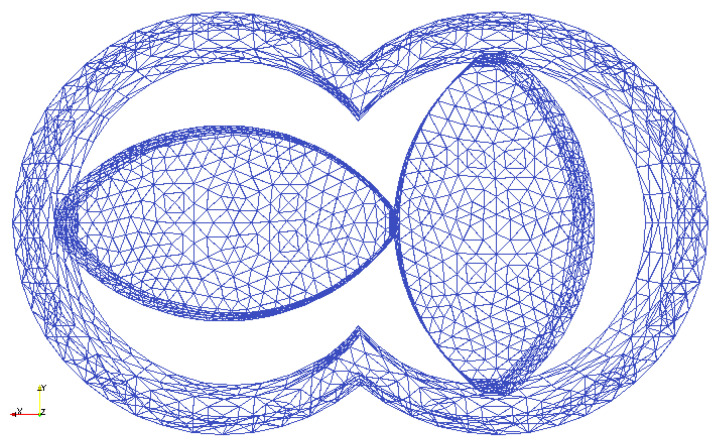
The 3D STL mesh for the kneading discs and barrel used for TSG mixing zone simulation.

To investigate the mixing of granulation liquid and formulation powder in the mixing zone of a TSG, DEM simulations were performed using the approach discussed in Section 2 and knowledge gained from the base case of the simple periodic simulation box (Section 2.3.1). A 3D CAD model for the kneading discs and barrel were created in SALOME and the triangulated surface mesh (STL) was created using Gmesh. The geometry was based on the screw design which is used in the TSG of the ConsiGma-25 unit (GEA Pharma Systems, Collette™, Wommelgem, Belgium) (Figure 3). The screws were rotated at a fixed speed. Approximately 46,000 mono-disperse particles of diameter $d_{p}$ and density $\rho_{p}$ were placed in the free space between the fixed-mesh of barrel and the rotating-mesh of two kneading discs. A periodic interface was created for the axial boundaries of the mixing section to allow particles that exit the section to enter from the other side. This periodic interface avoids to have walls as axial boundaries that would induce potential negative effects (e.g., wall layering).

## 3. Results and Discussion

### 3.1. Solid-Liquid Mixing in the Simple Periodic Simulation Box

**Figure 4:**
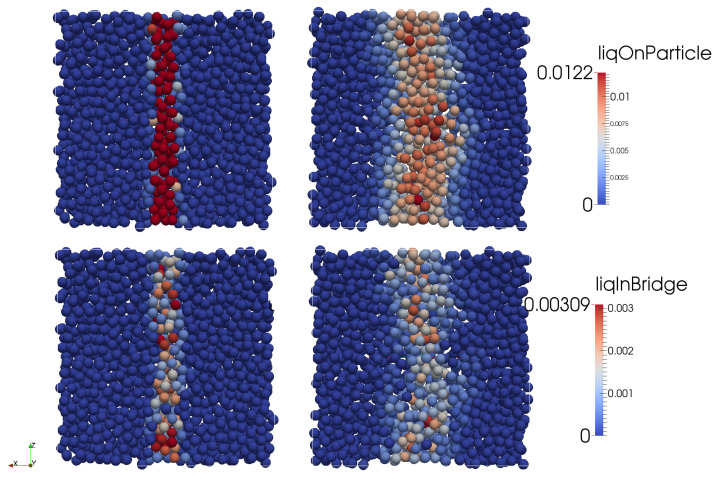
Transfer of liquid from particle surface (**top left**) to other particles by convective transport (**top right**) and transfer of liquid from particle surface to liquid bridges between particles by conductive transport before (**bottom left**) and after shearing (**bottom right**).

The solid-liquid mixing in a dense particle system was first investigated by the DEM simulation for a sheared assembly of particles discussed in Section 2.3.1. Shearing of particles resulted in (i) convective transport of liquid by particle motion (observed as liquid distribution), and (ii) the transfer of liquid from the particle surface to the liquid bridges (and vice versa) which is considered as conductive transport. The latter transport mechanism also results in the formation of agglomerates (Figure 4).

To investigate the effect of changes in the bulk density and fill ratio of the powder bed inside a TSG barrel, the volume fraction of the particles was changed in the simulations. To investigate the effect of liquid addition rate during granulation, the initial liquid loading on the particle in the wetting zone was changed in the simulations. The effect of liquid addition method at one point or two points, which basically affects the width of the addition zone, was investigated by changing the width of the wetting zone. The response of all these investigations on mixing and granulation was recorded in terms of change in the average number of liquid bridges per particle ${\bar{Z}}_{b}$ and the fractional amount of liquid in bridges $V_{bf}$. An increase in ${\bar{Z}}_{b}$ indicates an increasing aggregation level. Normally, the trends for ${\bar{Z}}_{b}$ and $V_{bf}$ should follow the same pattern. However, a change in their relation indicates the dominance of convective or conductive transport during wet granulation. Moreover, an increase in the screw speed leads to an increase in the shear level, which was investigated by increasing the applied shear to the particle assembly in the shear box.

#### 3.1.1. Effect of Change in Volume Fraction of Particles

**Figure 5:**
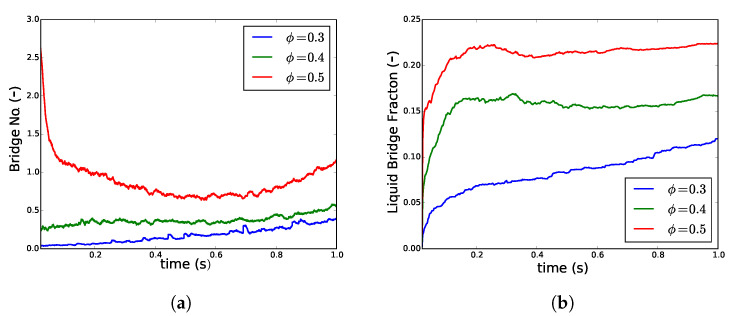
Changes in (**a**) average number of liquid bridges per particle ${\bar{Z}}_{b}$ and (**b**) the volume fraction of liquid in bridges $V_{bf}$ when the particle volume fraction ϕ was increased from 0.3 to 0.5 for liquid loading of 7.5 × 10^−4^.

The filling ratio inside the TSG varies significantly due to change in the screw speed, material throughput, and flow restriction by the kneading elements. In order to understand the effect of change in the fill ratio using simulations, the volume fraction of the particles in the simple periodic shear box was varied in the range of 0.3 to 0.5. Increasing the fill ratio resulted in a higher number of particles in the wetting zone (Figure 2). This is directly reflected by more liquid bridges for the high fill ratio at the beginning of the simulation (Figure 5a). However, as the shearing progressed ${\bar{Z}}_{b}$ increased for conditions with lower volume fractions (ϕ=0.3,0.4) whereas for the high volume fraction (ϕ=0.5) it first reduced and then equilibrated to gain some increase. The volume fraction of liquid in bridges $V_{bf}$ also showed the same patterns where the liquid bridge fraction was higher for higher ϕ, and it always remained so compared to the lower ϕ despite shearing. However, while $V_{bf}$ equilibrated as shearing progressed for higher volume fraction (ϕ=0.4,0.5), it kept on increasing for low volume fraction (ϕ=0.3). Additionally, at a high fill fraction, while ${\bar{Z}}_{b}$ was lowering by initial shearing, $V_{bf}$ increased. This indicates shear elongation, thus more liquid in the capillary bridges followed by breakage of these bridges.

The results indicate that with a high fill ratio, the (bridge and agglomerate) breakage mechanisms dominate over agglomeration, which results in redistribution of liquid. However, at a low fill ratio, agglomeration is the principal mechanism of progress in granulation. In order to keep both agglomeration and breakage as the active mechanism of granulation during simulation, hereafter ϕ has been fixed to intermediate level (ϕ = 0.4) unless stated otherwise.

#### 3.1.2. Effect of Change in Liquid Loading on Particles

**Figure 6:**
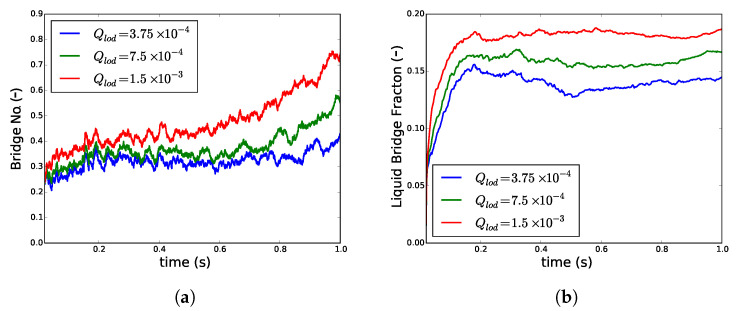
Changes in (**a**) average number of liquid bridges per particle ${\bar{Z}}_{b}$ and (**b**) volume fraction of liquid in bridges *V*_*bf*_ when the liquid loading on particles *Q_lod_* was increased at particle volume fraction *ϕ* of 0.4.

In a TSG, the amount of liquid loaded on the first wetted particles in the wetting zone changes when liquid addition rate or the fill fraction is changed. The shear mixing distributes this differently liquid loaded particle to proceed to a certain granulation end-point. This was simulated by increasing the liquid loading of the particles in the wetting zone $Q_{lod}$ (Equation (10)). The effect of an increase in liquid loading resulted in an increase in both ${\bar{Z}}_{b}$ and $V_{bf}$ (Figure 6). At a low $Q_{lod}$ (3.75 $\times \;10^{-4}$), after an initial increase, ${\bar{Z}}_{b}$ equilibrates at a certain level and later started to increase (Figure 6a). However, at higher levels of $Q_{lod}$ (7.5 $\times \;10^{-4}$, 1.5 $\times \;10^{-3}$) ${\bar{Z}}_{b}$ kept on increasing. Interestingly, although $V_{bf}$ had already achieved equilibration (Figure 6b), the ${\bar{Z}}_{b}$ was increasing. The ${\bar{Z}}_{b}$ increased further at high shear and this elevation was more at higher $Q_{lod}$.

This indicates that when more liquid is available, the liquid on the particle surface gets rapidly transferred to the liquid bridges. At low liquid loading, in the attempt to form more bridges by changing rearrangement, some liquid is returned back to the particle surface. However, in particles with higher loading, the initial liquid bridges rearranged themselves to form more bridges without giving away the liquid to the particle surface.

#### 3.1.3. Effect of Change in Liquid Addition Zone Width

The width of the wetting zone in a TSG and its effect on the effective distribution of liquid inside the barrel is an important aspect of solid-liquid mixing inside the granulator. This becomes especially crucial in the case of TSG because granulators from different manufacturers come with one or two liquid addition ports, which should influence the wetting zone. Varying the width of the wetting zone in the granulator while keeping the liquid flux constant allowed investigation of this effect.

**Figure 7:**
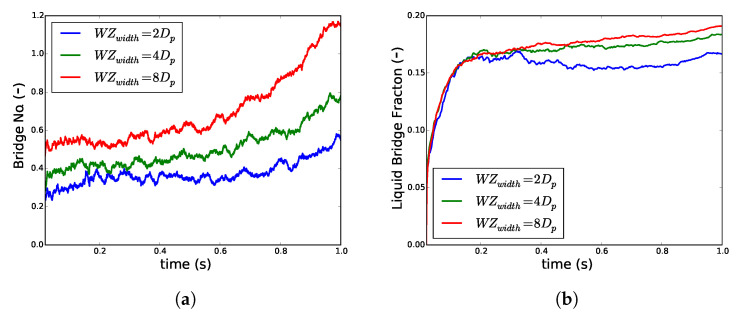
Changes in (**a**) average number of liquid bridges per particle ${\bar{Z}}_{b}$ and (**b**) volume fraction of liquid in bridges *V_bf_* when the wetting zone width *WZ_width_* was increased, keeping the flux of liquid addition constant and particle volume fraction *ϕ* of 0.4.

Despite a drastic increase in the wetting zone width $WZ_{width}$ from 2$d_{p}$ to 8$d_{p}$, the pattern of increase in the liquid bridge fraction remained essentially the same (Figure 7). However, as the shearing time increases, in the case of a narrower wetting zone, the liquid from the bridge is given back to the particle surface, indicating a dominance of convective transport of liquid (Figure 7a). When the wetting zone was wide, there was a slight increase in ${\bar{Z}}_{b}$ indicating an increase in the volume of liquid in bridges. This also translated in $V_{bf}$ but the effect was delayed. While ${\bar{Z}}_{b}$ increased initially, $V_{bf}$ showed a very limited increase for a narrower wetting zone (Figure 7b). However, the ${\bar{Z}}_{b}$ increased when the $V_{bf}$ was already equilibrated for all the three different wetting zone widths. This increase was more pronounced for the larger wetting zone.

These results once again suggest that the liquid on the particle surface is quickly transferred to the liquid bridges. However, the liquid in bridges is later redistributed during the rearrangement of particles due to the shear to form more liquid bridges. The results also suggest that the liquid added to a broader area provides a clear benefit in terms of formation of more liquid bridges per particle, which is beneficial for granulation.

### 3.2. Solid-Liquid Mixing in the Mixing Zone of a TSG

**Figure 8:**
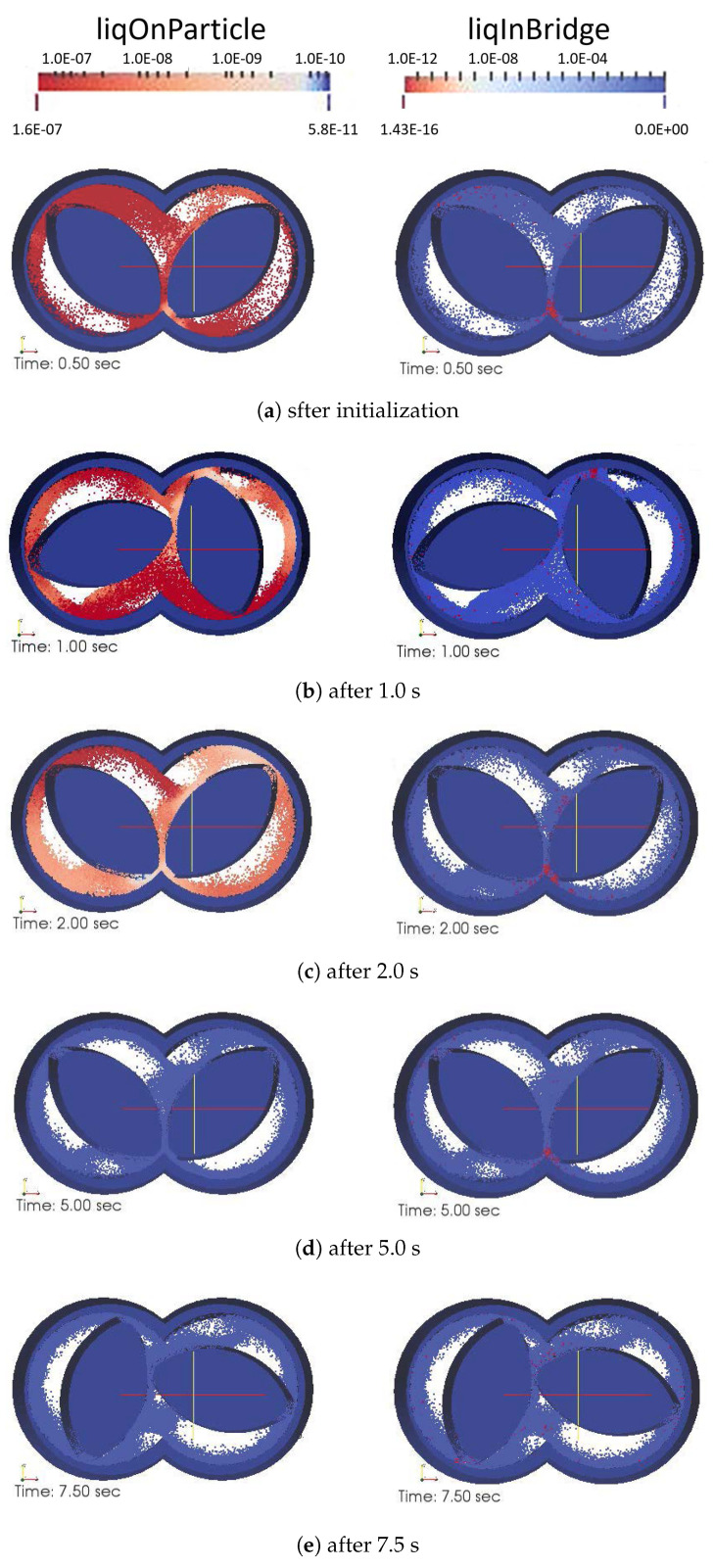
Changes in volume of liquid on particle surface (left side snapshot in each sub-plot) and liquid in bridges (right side snapshot in each sub-plot) when two kneading discs were co-rotating at 100 rpm. The plots below every snapshot indicate the volume of liquid on particle and volume of liquid in bridge for each particle in the system.

**Figure 9:**
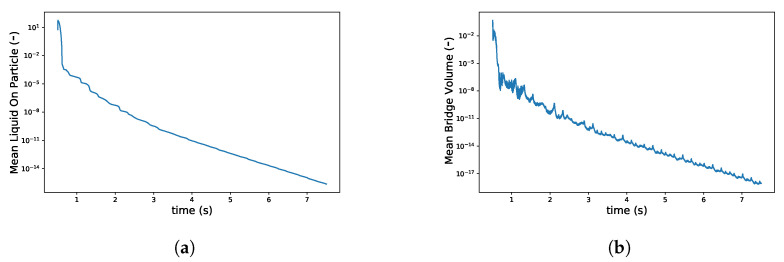
Changes in (**a**) volume of liquid on particle surface and (**b**) liquid in bridges when two kneading discs were co-rotating at 100 rpm.

To investigate the solid-liquid mixing in the mixing zone of a TSG, DEM simulations were performed using the set-up presented in Figure 3. About 46,000 particles were introduced, and the simulation was initialized for 0.5 sec after the wetting of particles (but not allowing any liquid transfer) exposed to a periodic interface in the *z*-direction to obtain representative randomness of particles (Figure 8a). After that, the amount of liquid on the particle surface and its transfer to liquid bridges by the shear mixing due to co-rotation of the two kneading discs was tracked in time. The shear mixing of particles resulted in a quick transfer of liquid from the wetted particle surface to neighboring dry particles in the barrel. This resulted in a rapid reduction in the liquid volume per particle over time from 0.15 to 2 $\times \;10^{-6}$ (see in Figure 8a–c and Figure 9a). The liquid on the particle surface was mostly transferred to the liquid bridges when the wetted powder was compressed between the closely inter-meshing kneading discs (see the right snapshots in Figure 8a, where particles with the maximum volume of liquid in bridge, i.e., particles connected with a higher number of liquid bridges are located in inter-meshing zone).

As granulation proceeds in TSG, both volumes of liquid on the surface of particles and the liquid in primary liquid bridges also reduced in this period (Figure 9b). This suggests that liquid transferred from particle surfaces was not contributing to agglomeration as a first step, but that conductive transport of liquid from the particle surface to liquid bridges between adjacent particles is the dominating first step to induce agglomeration. Hence, the primary liquid bridges were mostly present in over-wetted lumps, from which liquid was then convected under the shearing motion leading to the formation of newer liquid bridges. Further shear mixing by the kneading discs resulted in the equilibration of liquid on the particle surface and an increase in the number of liquid bridges. A simultaneous transfer of liquid from particle surface to liquid bridges led to oscillation in the mean liquid bridge volume. However, due to liquid mass conservation across particle surface and liquid brides, the liquid bridges contained a lower amount of liquid as time progressed and their number increased.

The results from this study suggest that the agglomeration is a delayed process, which is a follow-up event of equilibrated wetting of the particle. The convective mixing between solid-liquid followed by conductive transport of liquid from particle surface in the mixing zone leading to agglomeration confirms the importance of understanding the transport and mixing within a TSG. The results from this study are preliminary and require a more detailed study to understand the liquid distribution in other zones of the TSG, as well as the impact of screw geometry. Nevertheless, this modeling technique can be very useful for a detailed investigation of the mixing of solid particles and liquid. Since every particle in the simulation is tracked, the velocity and contact frequency information can be extracted to be used in refined PBE-based models for a more mechanistic simulation with local insight.

## 4. Conclusions

This study presented a theoretical analysis of the particle and liquid mixing based on particle-scale DEM simulations, in the context of HSWG using TSG. In the initial simulations, individual particles in a short quasi-two-dimensional simulation domain were tracked. Liquid bridge formation, rupture, as well as the effect of the bridges on inter-particular forces were simulated. Thereafter the same simulation approach was applied to the mixing zone of a TSG and the effect of process parameters on the particle and liquid mixing rate has been investigated. The results from this study demonstrate the potential of the particle-scale DEM method in modeling solid-liquid mixing and developing a detailed understanding of relevant phenomena. An extension of this study should be performed for detailed investigation of solid-liquid mixing along the complete length of a TSG. Since every particle in the simulation is tracked, the velocity and contact frequency information can be extracted to be used in PBE-based models for more mechanistic, still computationally realistic simulations. Moreover, there are multiple opportunities to increase the fidelity of the DEM simulations in the future, e.g., by considering viscous liquid bridge forces, or surface roughness effects.

## Figures and Tables

**Table 1:** Summary of input parameters used in particle-scale modeling.

Quantity	Symbol	Value	Unit
Particle diameter	$d_{p}$	1.00E-03	[m]
Young’s modulus	*G*	3.45E+9	[N/m^2^]
Initial particle velocity	$v_{x}$, $v_{y}$	1, 0.1	[m/s]
Coefficient of restitution	$e_{n}$	0.9	[–]
Coefficient of friction	µ	0.1	[–]
Poisson ratio	ν	0.33	[–]
Film thickness	$h_{0}$/$r_{\mathrm{eff}}$	1.00E-02	[–]
Dimensionless filling rate coefficient	$c_{f}$	1	[–]

## Abbreviations

The following abbreviations are used in this manuscript:

**List of Acronyms**	
DEM	discrete element method.
HSWG	high shear wet granulation.
PBE	population balance equation.
PBM	population balance model.
TSG	twin-screw granulator.
**List of Symbols**	
$t_{c}$	Characteristic contact time [s].
$c_{f}$	Dimensionless filling rate coefficient [−].
δ	Normal overlap between particles *i* and *j* [m].
$d_{p}$	Particle diameter [m].
ϵ	Dimensionless reference film thickness [−].
$\eta_{n}$	Normal damping coefficient [Ns/m].
$e_{n}$	Normal coefficient of restitution [−].
$\eta_{t}$	Tangential damping coefficient [Ns/m].
$F_{i}^{b}$	body force of a particle *i* [N].
$F_{ij}^{coh}$	Cohesion force between particle *i* and *j* [N].
$F_{ij}^{con}$	Contact force between particle *i* and *j* [N].
$F_{i}^{c,n}$	Normal component of force acting on particle *i* [N].
$F_{i}^{c,t}$	Tangential component of force acting on particle *i* [N].
γ	Shear rate [1/s].
$\gamma^{*}$	Scaled shear rate [−].
$h_{0}$	Dimensional reference film thickness [m].
$k_{n}$	Normal spring stiffness [N/m].
$k_{t}$	Tangential spring stiffness [N/m].
$L_{p}$	Volume of liquid present on the particle $\left[m^{3}\right]$.
$L_{p,0}$	Reference liquid content on the particles [−].
$m_{\mathrm{eff}}$	Effective mass of the particle [kg].
$\mu_{l}$	Dynamic viscosity of liquid $\left[\mathrm{Ns}/m^{2}\right]$.
$n_{b,i}$	Number of liquid bridge connected to particle *i* [−].
$n_{p}$	Number of particles [−].
$n_{p,liq}$	Number of particles in the liquid addition region [−].
$n_{ij}$	Unit normal vector [−].
ω	Eigen frequency of damped harmonic oscillator [1/s].
ϕ	Volume fraction of particles [−].
$\phi_{tr}$	Fraction of liquid on the surface that is transferred into the bridge [−].
$Q_{i}$	Liquid addition rate to particle *i* in the liquid addition region $\left[m^{3}/s\right]$.
$Q_{lod}$	Dimensionless liquid load per particle [−].
$Q_{tr}$	Liquid transfer rate for particle $\left[m^{3}/s\right]$.
*r*	Radius of the particle [m].
$r^{*}$	Position vector of the particle [m].
$r_{\mathrm{eff}}$	Effective radius of the particle [m].
$\rho_{p}$	Density of the particles $\left[\mathrm{kg}/m^{3}\right]$.
$\sigma_{l}$	Surface tension of liquid [N/m].
$t_{\exp}$	Liquid addition time [s].
$t_{\mathrm{ref}}$	Reference liquid bridge filling time [s].
$u_{ij}^{t}$	Tangential overlap between particles *i* and *j* [m].
$V_{b}$	Liquid bridge volume $\left[m^{3}\right]$.
$V_{bf}$	Liquid bridge fraction [−].
$v_{ij}^{n}$	Normal relative particle velocity components [m/s].
$v_{ij}^{t}$	Tangential relative particle velocity components [m/s].
${\bar{Z}}_{b}$	Average number of liquid bridges per particle [−].
